# Impact of Housing Environment on the Immune System in Chickens: A Review

**DOI:** 10.3390/ani10071138

**Published:** 2020-07-05

**Authors:** Tanja Hofmann, Sonja S. Schmucker, Werner Bessei, Michael Grashorn, Volker Stefanski

**Affiliations:** 1 Department of Behavioral Physiology of Livestock, Institute of Animal Science, University of Hohenheim, Garbenstr, 17, 70599 Stuttgart, Germany; sonja.schmucker@uni-hohenheim.de; 2 Department of Livestock Population Genomics, Institute of Animal Science, University of Hohenheim, Garbenstr, 17, 70599 Stuttgart, Germany; werner.bessei@uni-hohenheim.de (W.B.); michael.grashorn@uni-hohenheim.de (M.G.)

**Keywords:** poultry, chicken, immune system, housing condition, stress, stressors, immune modulation, health, housing form, light regime

## Abstract

**Simple Summary:**

In poultry production, chickens are confronted with a wide range of potential stressful conditions including restricted movement, long light phases or poor air quality. It is well known that stressors can have negative effects on the immune system. A fully functional immune system is, however, not only essential for chicken health and welfare but also for high productivity and safe animal products. This review summarizes current knowledge about the impact of housing form, light regime, aerial ammonia and hydrogen sulfide concentrations on the immune system in chickens and outlines possible mechanisms and interactions.

**Abstract:**

During their lifespan, chickens are confronted with a wide range of acute and chronic stressors in their housing environment that may threaten their welfare and health by modulating the immune system. Especially chronic stressful conditions can exceed the individual’s allostatic load, with negative consequences for immunity. A fully functional immune system is mandatory for health and welfare and, consequently, also for high productivity and safe animal products. This review provides a comprehensive overview of the impact of housing form, light regime as well as aerial ammonia and hydrogen sulfide concentrations on the immune system in chickens. Certain housing conditions are clearly associated with immunological alterations which potentially impair the success of vaccinations or affect disease susceptibility. Such poor conditions counteract sustainable poultry production. This review also outlines current knowledge gaps and provides recommendations for future research.

## 1. Introduction

In recent decades, the poultry industry was optimized for highly efficient and cost-sensitive egg and meat production, leading to major changes in management practices, with specialized and intensified production processes. However, public concern about the negative impact of intensive production on animal welfare and food safety has also markedly increased [1]. Animal health is an integral part of welfare and a prerequisite for both high productivity and safe products for human consumption [2,3]. Essential for good health is a fully functional immune system, which, however, may be threatened by stressors and other adverse environmental stimuli. Various factors like housing, temperature, air quality or light regime may act as stressors, with potentially negative effects on the immune system [3,4]. This review provides a comprehensive overview of possible consequences of common housing conditions on the immune system in poultry. Furthermore, we identify current knowledge gaps and offer recommendations for future research.

## 2. Brief Overview of the Avian Immune System

In addition to the many similarities with the mammalian immune system, the avian immune system contains several particularities. Birds’ immune system consists of an innate and an adaptive arm, both including cellular and humoral components. Avian heterophils are functionally homologous to mammalian neutrophilic granulocytes, as they show strong phagocytic activity and are the first cells to be involved in inflammatory responses [5]. The avian repertoire of granule components, however, differs from their mammalian counterparts. Chickens have more basophils than mammals, which play a central role in early inflammation and immediate hypersensitivity [6]. Avian eosinophils are associated with allergic reactions and parasitic infections, but in contrast to mammalian eosinophils, also serve as early modulators of inflammation in delayed-type hypersensitivity responses [7]. A major difference to mammals is that avian erythrocytes and thrombocytes are nucleated. Furthermore, thrombocytes also show phagocytic activity, including killing of bacteria, due to their ability to produce an oxidative burst [8]. Like in mammals, dendritic cells in chickens play a central role as antigen-presenting cells, presenting antigens in the context of the major histocompatibility complex to lymphocytes and thus initiating adaptive immune response [9]. Birds’ adaptive immune systems comprise all major lymphocyte subsets like T lymphocytes, B lymphocytes and natural killer cells. However, birds have larger numbers of circulating γδ T cells compared to humans or rodents [10]. B lymphocytes develop in a unique avian organ, the bursa of Fabricius, and not in the bone marrow as in mammals. Furthermore, the repertoire of B lymphocyte receptors and antibodies is more limited, as the generation of antibodies in birds is based only on a single copy of genes for V and J segments for both the light and heavy chains. A diversity of antibodies is produced by gene conversion rather than gene recombination [11,12]. Unlike mammals, birds are also not able to generate new antibody receptor molecules throughout their lifespan [12]. So far, only three classes of immunoglobulins (Ig)—IgM, IgY and IgA—have been identified in chickens, and all differ in structure from the mammalian homologs. Avian IgM is functionally similar to that of mammals and is the predominant isotype after exposure to a new antigen. IgY, the homolog to the mammalian IgG, is similarly the predominant antibody during secondary antibody response and infection but lacks any subtypes that appear in mammals. IgA is found in mucus and secretions. So far, no avian homologs of IgE and IgD have been described, but IgY shares certain properties with mammalian IgE [12]. Recently, natural antibodies were shown to be present in chickens [13]. Natural antibodies are defined as antigen-binding antibodies present in individuals without previous exposure to the recognized antigen. As such, they serve as the first line of defense, likely contributing to disease resistance by the enhancing humoral adaptive immune response [14].

While the genome region encoding the major histocompatibility complex is highly polymorphic in mammals, it is minimal in birds, containing only two class I and two class II genes [15]. Furthermore, chickens’ toll-like receptors differ at the level of ligand specificity, the formation of receptor complexes, and activated signaling pathways [16].

Cytokines act as chemical messengers and affect the proliferation, differentiation and activity of immune cells [17]. Many functional homologs of mammalian major cytokines like interleukin (IL)-1β, IL-6, IL-12, IL-2, interferon (IFN)-γ, IL-4, IL-10 [17,18] and recently also tumor necrosis factor (TNF)-α [19] are described in chicken. However, cytokine families generally comprise a smaller number of genes in chickens compared to mammals, and in most cases their precise biological function is still unknown [18]. In poultry, cytokines are routinely measured by quantitative polymerase chain reaction, which quantifies the expression of cytokines on a mRNA level. To quantify secreted cytokines on a protein level, measuring the amount of functional cytokines would be preferable; however, investigation is hampered by the lack of a proper cytokine ELISA. Distinct differences also become evident in comparison with the mammalian lymphoid system. Most birds, including chickens, lack capsulated lymph nodes, the primary site of antigen presentation in mammalian species. Hence, the emergence and induction of the adaptive immune response occurs locally to the site of infection in the many lymphoid aggregates found in the chicken’s mucosal tissues. Mucosa-associated lymphoid tissues are well developed in chickens and are found in numerous locations, especially in the gastrointestinal tract, including some unique avian structures like the Meckel’s diverticulum and the cecal tonsils [20,21].

The particularities of the avian immune system are described in detail in several reviews [10,22,23,24,25].

## 3. Stress and Neuroendocrine–Immune Interactions

During their lifespan, chickens are confronted with a wide range of acute and chronic stressors potentially threatening their welfare and health [26]. As in other vertebrate species, stressors activate the hypothalamic–pituitary–adrenal axis and sympathetic–adrenal–medullary axis in birds, resulting in the release of glucocorticoids as well as catecholamines. Both stress systems work together to enable a successful adaption to a challenging environment on a behavioral and physiological level [27]. The predominant glucocorticoid in birds is corticosterone (CORT) [28]. Like in mammals, glucocorticoids and catecholamines bind to specific receptors that are expressed in almost every tissue in the chicken, including the brain, pituitary, lung, liver, spleen, ovary, testes and the bursa of Fabricius [29,30]. In mammals, glucocorticoid receptors are differently expressed on immune cells, with lymphocytes usually expressing higher levels of glucocorticoid receptors than granulocytes [31,32,33]. In contrast, adrenergic receptors are highly expressed on cells of the innate immune system [34]. Both glucocorticoids and catecholamines have strong immunomodulatory properties in mammals [35,36,37,38,39,40,41,42,43] and birds [44,45,46,47,48].

After exogenous CORT administration, plasma CORT levels were elevated and peripheral heterophil count increased while peripheral lymphocyte count decreased. As a consequence, changes in the ratio of heterophils to lymphocytes occur [49,50]. Furthermore, CORT administration indicated changes in heterophil size, shape, and granulation, as well as in lymphocyte cytoplasmic characteristics [50]. This reaction represents an evolutionarily conserved adaptive response that might contribute to an enhancement of the immune surveillance by redistributing leukocytes among compartments, blood, lymphoid and non-lymphoid tissue [51]. The ratio of heterophils (or neutrophils) to lymphocytes (H/L) is, therefore, widely accepted as a tool to assess stress in all vertebrates [52]. CORT administration was also shown to up-regulate cytokine mRNA expression of pro-inflammatory cytokines and chemokines [53,54,55] and to suppress lymphocyte proliferation in chickens [45,47]. Chronic and repeated exposure to CORT caused a sustained elevation in plasma CORT concentrations and the H/L ratio as well as a decrease in the relative weight of immune organs and in the antibody response to infectious bronchitis virus (IBV) vaccination [45,49,55].

It is well known that acute stressful conditions often have promoting effects on innate immunity, while especially chronically elevated glucocorticoid levels can exceed an individual’s allostatic load, with negative consequences for health [56,57,58,59]. It is obvious that the neuroendocrine and immune system interact to maintain homeostasis when an organism is under severe or chronic stress. Normally, a feedback system protects the organism by down-regulating neurotransmitters, hormones and cytokines that act as mediators. However, chronic stressful conditions can overwhelm an individual’s allostatic load, having negative consequences on immunity [40].

However, animals differ markedly in their behavioral and physiological stress responses (coping styles). Two major coping styles exist: birds with a proactive coping style show more active responses to challenging situations, while a reactive coping style is associated with immobility and withdrawal [60,61]. Furthermore, reactive birds have higher CORT concentrations and H/L ratios than proactive birds [62,63]. Pusch et al. [64] also demonstrated a higher inflammatory response to phytohemagglutinin (PHA) injection in reactive chickens but no difference in phagocytic activity or antibody response, indicating that reactive individuals also differ in immune responses.

## 4. Impact of Housing Systems on the Immune System in Chickens

Under commercial housing conditions, chickens are kept either in cage systems or alternative systems. Housing forms mainly differ with respect to group size, freedom of movement and complexity of the environment [65]. Cage systems are subdivided into conventional cages (CC) and enriched cages (EC). CC lack adequate space for movement and elements such as nests, perches and litter to allow for behavioral expression. EC are similar to CC with regard to production efficiency and hygiene, but do also not allow for full behavioral expression due to the restricted space allowance [58,59]. Alternative housing systems include indoor loose housing systems (i.e., single-tier and multi-tier floor pens (FP)) and outdoor loose housing (i.e., free range (FR) and organic systems). In this case, birds are kept in larger groups with sufficient space for performance of a full repertoire of natural behavior, with access to a large litter area, nests and perches and, in the case of FR, also outdoor space [65]. Worldwide, CC are still the predominant housing form for commercial egg production. In contrast, broilers are reared mainly in littered floor pens [66]. In order to improve animal welfare, alternative housing systems are however becoming increasingly popular [66,67]. In general, birds kept in alternative systems can express their full natural behavior repertoire and display more comfort and activity behavior than in cage systems. On the other hand, birds kept in alternative systems show more aggression, feather pecking, cannibalism and a higher incidence of diseases [66,67]. The influence of different housing systems on behavior, performance and some general health parameters has already been reviewed in earlier studies [65,66,67,68,69].

Most studies on the effect of different housing systems on the immune system in chickens are focused on comparisons between CC and EC (Table 1). With respect to immune cell numbers, the outcome is not consistent. While some reports found higher relative numbers of heterophils [70,71,72], monocytes and eosinophils [72] as well as lower numbers of lymphocytes [70,71,72] in hens in CC than in EC, other reports could not confirm differences between both housing forms on respective immune cell types [70,73,74]. However, most studies that included the H/L ratio reported higher ratios in CC hens [71,72,73,75], which is most likely the result of anti-parallel shifts in heterophil and lymphocyte numbers. With respect to the functionality of immune cells, a diverging picture emerges as well. Matur et al. [70,71] reported a lower antibody response to sheep red blood cells (SRBC) in hens in CC compared to EC, suggesting a lower adaptive humoral immunity. Other studies, however, did not find any differences with respect to antibody response as a consequence of vaccination against Newcastle disease virus (NDV) [72,74], IBV [72] or to basal plasma IgY concentrations [76]. No differences between CC and EC were reported with respect to the functionality of heterophils and monocytes [70,71,75] or lymphoid organ weights such as thymus, bursa and spleen [70,71].

Nevertheless, more obvious immunological differences between hens in CC or EC became evident when animals were stressed by mixing or transport. The number and functionality of heterophils (oxidative burst and chemotaxis) did not respond to stress in CC, but were increased in stressed hens in EC [70,71], while relative numbers of lymphocytes were lower [70]. Moreover, the relative proportion of T helper (TH) cells and cytotoxic T cells (CTL) among blood lymphocytes was higher in stressed EC [70,71]. Taken together, these findings point to a stronger responsiveness of the innate immune system in hens in EC under acute stress conditions.

At this point, it can be concluded that not all studies found immunological differences between hens kept in EC or CC, but if differences were detected, they indicate a higher stress load in hens in CC due to the higher H/L ratio.

A smaller number of studies compared the immune status of hens in cage and alternative systems (FP and FR), again with diverging results. With respect to cell numbers, hens reared in alternative systems show lower relative numbers of heterophils [72,73,77] and higher numbers of lymphocytes [72,73], an effect reflected by lower H/L ratios under alternative conditions [72,73,75,77,78]. Some authors, in contrast, reported no differences in the relative proportions of heterophils [72,79], monocytes, eosinophils and basophils [72,73,79] or lymphocytes [77,79] between alternative and cage housing (both CC or EC). The findings are moreover not uniform with respect to immune functioning. Some studies found evidence for a down-regulated adaptive immune function in hens in CC compared to hens in FR, as indicated by lower peripheral concanavalin A (ConA)-induced lymphocyte proliferation [80] and lower specific antibody titer after vaccination against NDV [81,82] or IBV [72,81]. However, other reports did not find any differences in antibody concentrations after lipopolysaccharide (LPS) stimulation [80] or after vaccination against IBV or infectious bursal disease virus [82]. Van Loon et al. [80] even found higher antibody concentration and lymphocyte proliferation after KLH (keyhole limpet hemocyanin) stimulation in CC hens compared to FR. No difference in the functionality of phagocytes was reported [75,79,80].

Studies comparing alternative systems (FP vs. FR) were rarely conducted. Diktas et al. [83] found no difference for the H/L ratio and relative numbers of peripheral leukocyte subsets while Campo et al. [84] found higher H/L ratios in birds in FP compared to FR. Furthermore, a higher ConA-induced lymphocyte proliferation and lower antibody concentration after KLH stimulation were found in hens in FR compared to FP [80].

Data from endocrine and behavioral studies revealed higher CORT concentrations and tonic immobility (TI) reaction in hens in CC compared to EC [85]. Alternative systems were shown to induce higher CORT concentrations compared to cage systems [78,86,87]. However, some authors also did not find differences between housing forms regarding stress hormone concentration and fear response [73,74].

We can conclude that some designs of housing systems might reflect stressful conditions in hens. This stress can modify a hen’s immune system and, therefore, affect vaccination response or disease susceptibility. Nevertheless, the particularities of specific management conditions still need to be investigated in more detail. With respect to alternative systems, results are apparently more complex and no unanimous conclusions regarding their stress load can currently be deduced from the literature.

### Effect of Housing Systems on the Immune System—Possible Mechanisms and Conclusions

What are the possible underlying factors for the effect of housing form on the immune system in chickens? One major difference between CC and other housing scenarios is a reduced environmental complexity; CC hens in particular show behavioral, endocrine and immunological signs of stress. In general, environmental enrichment is known to reduce housing stress in birds [26,88,89], and the observation of higher H/L ratios in hens in CC supports this view. Moreover, Campderrich et al. [26] demonstrated that laying hens in a complex environment have a better inflammatory response and higher lymphocyte proliferation when exposed to cold stress. A recent review by Campbell et al. [90], with focus on environmental enrichment during rearing on the behavioral and physiological development of laying hens, also concluded that stress-reducing effects of enrichment are associated with a positive effect on immune competence.

Another aspect of enriched housing environments is that they allow for increased physical activity [75,86]. It has been amply demonstrated in mammals that physical activity positively influences the immune system by reducing stress hormone concentrations. Moreover, it increases the cytotoxicity of T lymphocytes and natural killer cells, the phagocytic activity of neutrophils and macrophages, and the vaccination response [91]. Hens in CC, having only limited space for locomotor activity, show an impaired immune function. Quite obviously, further studies are required to link physical activity and immune function with chicken housing systems. Another contributing factor is the hygiene status of the housing form, which may affect the immune status under enriched conditions. Higher exposure to excrements under enriched conditions, such as deep litter and free range systems, may result in an increased load of bacteria and fungi [65]. It is well known that the presence of microorganisms activates heterophil functions [92].

In conclusion, chickens in the above-cited studies were faced with various social and environmental challenges. This diversity may well represent the variation in housing and management factors under practical conditions, but also makes any cross-study comparison difficult. For future studies on the effect of housing forms on the immune system, a higher degree of standardization would be desirable, e.g., with respect to group size and stocking densities, but also with regard to the genetic background of the animals. Obvious interactions between housing type and genotypes exist that affect the immunological outcome [78,80,81]. Current breeds were mainly selected for production under low levels of environmental stimuli but the outcome with respect to immune competence might differ considerably when birds are exposed to enriched environmental conditions [80].

## 5. Impact of Light Regime on the Immune System in Chickens

Light is one of the most important exogenous factors regulating physiological and behavioral processes as well as entraining circadian rhythms of hormones and immune cells in birds and mammals [93,94,95,96]. Since most chickens are housed indoors, birds are usually exposed to artificial rather than natural light. Light management of poultry focuses on three different light properties: photoperiod, light intensity and light color/wavelength [88]. Photoperiod manipulation is the most prominent aspect of light management in poultry production. Distinct light–dark cycles (L:D) enable the development of a circadian rhythm [97]. Broilers given continuous or near-continuous light develop no circadian rhythm, whereas they do under extended dark periods such as 12L:12D [98] or 16L:8D [99]. The development of a circadian rhythm is considered an important indicator of welfare in domestic animals [97], and its disruption is associated with a variety of metabolic and immune disorders [98]. Light programs are not standardized and vary between countries [96]. In the European Union, an uninterrupted darkness of 6 h (h) for broilers [100] and 8 h for laying hens [101] is mandatory. In most countries, broilers are exposed to continuous or near-continuous illumination for 24 L or 23 L:1 D to maximize feed intake and body weight gain [102,103]. However, extremely long light cycles are associated with reduced performance, decreased locomotion and increased health problems [96,102,104]. In addition to the common light programs with one phase each of continuous light and dark within 24 h (constant light = CL), intermittent lighting (IML) programs with alternating phases of short light and dark periods exist, like 4 L:4 D, 2 L:2 D, 1 L:2 D. These programs have been tested mainly under experimental conditions and are rarely used in practical broiler or layer production.

The color of light depends on the type of light source. In addition to conventional incandescent and fluorescent illuminants, light-emitting diodes are increasingly used in poultry houses. The spectrum of wavelength is highly variable in all types of illumination and all of them differ considerably from the spectral pattern of the light in the natural habitat of the birds [105]. In contrast to mammals with three single-cone photoreceptors, chickens have four types and differentiate wavelengths between 350 and 700 nm, i.e., they also perceive light on the infrared (longer wavelengths) and ultraviolet (shorter wavelengths) spectrum [106]. Studies report that shorter wavelengths (blue 450 nm, green 550 nm) have positive effects on broiler performance, while longer wavelengths (red 700 nm) increase the activity of broilers. The impact of light wavelength on welfare, behavior and performance of poultry was recently reviewed [107,108]. Due to the chickens’ good color vision, they may experience a better quality of vision in bright than in dim light. So far, the assessment of light intensity experienced by poultry is still based on parameters of the human perception of light. Due to the differences in anatomy and physiology of human and avian eyes, however, other parameters should be taken into account in order to optimize lighting conditions for chickens. Light intensity is commonly given as lux. Lux, however, does not consider UV-A light, even though these ultraviolet wavelengths contribute to brightness perception in birds [105]. Most modern light programs start with higher intensities during rearing (~20 lux), which are then decreased to 5 lux until the end of the growing period in broilers [96]. Light intensities ≥5 lux after the initial brooding period are said to stimulate metabolism and growth [104]. During the laying period, light intensities between 10 and 15 lux are recommended [109]. However, a minimum of 20 lux during photoperiod is compulsory in the European Union [100]. Low light intensities are associated with decreased activity and health [97]. A meta-analysis indicated that light intensities <5 lux lead to impaired foot health, light intensities <1 lux induce productivity loss, and light intensities >10 lux increase mortality and decrease the uniformity of the broilers. Furthermore, 30−200 lux light intensity is negatively related to body weight development and feed intake [110].

### 5.1. Photoperiod

Table 2 summarizes the influence of light constantly provided during long-day conditions (23 or 24 h of light) (LD-CL) or during short-day conditions (at least 6 h of darkness) (SD-CL). Various studies included in Table 2 reported an effect of the photoperiod on immune parameters. Under LD-CL conditions, total leukocyte numbers [111] and relative numbers of heterophils [112,113] were higher, whereas relative numbers of lymphocytes, monocytes, basophils and eosinophils were decreased [112,113]. Correspondingly, the H/L ratio was higher [112,113,114]. However, results are overall inconclusive, as other authors did not find any influence of photoperiod on the H/L ratio [115,116] or an even lower H/L ratio under LD-CL [111]. Kliger et al. [117] found a lower percentage of total T lymphocytes, TH cells and CTL under LD-CL, with no effect on B lymphocytes in the spleen of adult broilers. In contrast, the authors found reduced relative numbers of B lymphocytes and higher numbers of TH cells in younger broilers in LD-CL, indicating a different sensitivity to light dependent on age. With respect to the functionality of lymphocytes, findings are also inconsistent. Broilers under LD-CL showed lower pokeweed-mitogen (PWM)-induced proliferation of splenic lymphocytes [117] and specific antibody titers after SRBC stimulation in blood as well as delayed-type hypersensitivity to PHA and ConA [118]. In contrast, others did not report effects on the proliferation of peripheral lymphocytes [111,117], serum antibody titers against SRBC [103,111,114,119] or specific antibody titers after NDV vaccination [114]. An effect of the photoperiod on the weight of lymphatic organs was also not reported [114,120].

Whether light is provided constantly or intermittently might be another important factor in addition to variations in photoperiod, as already mentioned above. Below, we will consider the influence of constant light regimes under long-day conditions (LD-CL) as well as intermittent light regimes under short-day conditions (SD-IML) with at least 6 h of darkness. Most studies in Table 3 found differences regarding the immunological measures when both conditions were compared. Under LD-CL, lower absolute leukocyte numbers [111,121] and relative numbers of lymphocytes and monocytes [112] were reported, while relative numbers of heterophils were higher [112]. This anti-directional shift resulted in a higher H/L ratio under LD-CL in this report [112], while other studies did not find such an effect [111,122,123]. Kliger et al. [117] focused on splenocytes and found lower percentages of T lymphocytes, TH cells and CTL, but no difference in B lymphocytes under LD-CL in adult broilers compared to SD-IML. In young broilers, no difference in these parameters could be found [117], indicating again different sensitivity to light programs during different life stages. Most studies also found an effect of LD-CL or SD-IML on the functionality of immune cells. Broilers reared under LD-CL compared to SD-IML showed lower phagocytic activity of monocytes [124], mitogen-induced peripheral and splenic lymphocyte proliferation [111,117,125], as well as total serum IgM [126], plasma IgY [127], specific antibody titer after NDV vaccination [123] or SRBC stimulation [111]. Furthermore, the relative weight of bursa and thymus was decreased in broilers under LD-CL [124]. A few studies, however, did not find differences in the functionality of immune cells such as mitogen-induced peripheral lymphocytes proliferation [117,121], delayed-type hypersensitivity to PHA [125], total IgY or IgA [126], antibody titer after SRBC stimulation [103,122], pro-inflammatory cytokine production [121,125] or relative organ weight [123,124,126].

Again, immunological modulations by photoperiod may become obvious only in certain situations. After stressing with heat or LPS injection, birds under LD-CL showed a higher H/L ratio [122] but a lower total leukocyte count [121]. Moreover, ConA-induced peripheral lymphocyte proliferation [121], antibody titers after SRBC stimulation [122] and basophilic hypersensitivity response [125] were lower while pro-inflammatory cytokine concentration (IL-6) was higher in broilers under LD-CL compared to SD-IML [121,125].

Results comparing short-day light regimes given either intermittently (SD-IML) or constantly (SD-CL), are also inconsistent with respect to immune parameters. Some studies reported lower H/L ratios [111], higher total leukocyte counts [111], ConA-induced peripheral and splenic lymphocyte proliferation [111,117] as well as higher antibody titers after SRBC stimulation [111] in birds exposed to SD-IML. Other studies did not find any differences in the H/L ratio [112], relative numbers of peripheral [112] and splenic lymphocytes [117] or PWM-induced splenic lymphocyte proliferation [117] in broilers under SD-IML compared to SD-CL.

There are hints that CORT concentrations are higher in broilers kept under LD conditions [126], especially when they were additionally stressed [121,125]. Broilers also showed longer TI reactions under LD conditions, indicating higher fearfulness [114,116,123,128]. However, these parameters were not affected in other studies [103,111,112,119,129].

The overall conclusion from these studies is that birds kept under LD conditions have lower adaptive cellular and humoral immune responses than under SD conditions. Hence, keeping chickens under SD conditions could lead to a stronger responsiveness of the adaptive arm of immunity against bacterial infections and better responses to vaccinations. In addition, a light regime with IML could be used as an effective tool to specifically stimulate birds’ immune response. However, so far, it is not clear what is more important: the total hours of light and dark within a 24 h period, or whether light is given constantly or intermittently.

### 5.2. Light Color/Wavelength

There is evidence that the color of light affects the chickens’ immune system. Kim et al. [130] showed that broilers reared under white light have lower relative numbers of lymphocytes compared to red and yellow light, and a higher number of monocytes compared to green light, while the relative numbers of heterophils, eosinophils and basophils remained unaffected. Similarly, Gharahveysi et al. [131] reported no difference in the white blood cell count of broilers reared under green, yellow or red light, whereas the lowest H/L ratio was shown under red light, followed by blue-green, white and yellow-orange light [132]. Likewise, Archer [133] reported a lower H/L ratio in laying hens under red light compared to white light, while Hassan et al. [134] did not note a difference in the H/L ratio under different light colors. However, recent studies showed that UV light lowers the H/L ratio in laying hens [135] and broilers [136]. The number of intraepithelial lymphocytes and IgA+ cells in cecal tonsils and in the small intestine were shown to be higher under green and blue light compared to red light [137,138]. Studies investigating the functionality of immune cells emerge with a clearer picture. Broilers reared under blue or green light compared to red light showed higher ConA- and LPS-induced lymphocyte proliferation in blood [139,140], spleen [141], bursa of Fabricius [142] and thymus [143]. Likewise, the activation of macrophages was higher under blue and green compared to white light [144]. Furthermore, chickens housed under green or blue light were also shown to have higher antibody titers after NDV vaccination [139,140,145] and higher total plasma or serum concentrations of IgY and IgA in broilers [146]. However, in laying hens, no influence of light color on serum IgY concentration was reported [134]. In broilers, under green and blue compared to red light, the concentration of pro-inflammatory cytokine IL-2 was higher [139,140] and TNF-α lower [139]. It is interesting that the effect of light color also seems to be age dependent. The highest number of intestinal intraepithelial lymphocytes in broilers during the early growth stage was seen under green light, while numbers were highest under blue light during the late growth stage [138]. Similarly, green light showed the highest ConA- and LPS-induced lymphocyte proliferation in blood and spleen [140,141,144] and splenic IL-2 activity [141] during early growth stage, while at the end of the growth stage, blue light produced those same effects. Hassan et al. [146] reported higher serum IgY and IgA concentrations under yellow light compared to white light, with no difference when comparing yellow or white light with green or blue light in the early growth stage. In contrast, blue light promotes higher serum IgA concentrations than white light in the later growth stage, with no difference when comparing blue or white light with yellow or green light. Furthermore, nitric oxide production of splenocytes was higher in red light compared to blue, green and white light in mature but not in young broilers [141]. Nevertheless, some studies also show that there are differences between different blue and green shades [144,147], and colored lights do not always promote immunity compared to white light [140,141,143]. Moreover, exposing broilers to white light, red light, green light, and blue light during the early growth stage and then switching green light and blue light to blue light and green light, respectively, can have beneficial effects. The authors found elevated specific antibody titers after NDV vaccination, increased proliferation of peripheral T and B lymphocytes and increased IL-2 concentrations, but decreased TNF-α concentrations in the switched groups compared to the single monochromatic light groups. The authors, therefore, concluded that switching from green to blue (or blue to green) monochromatic light during development can promote immune response in broilers [139].

In summary, the studies show that shorter wavelengths like blue and green effectively enhance some immune functions, and that young birds are more responsive to green light and older birds to blue light.

Certain light colors also have a positive influence on animal welfare and stress load. Lower CORT concentrations were observed when chickens were housed under red light compared to white light [132,133] or if exposed to UV [135,136]. Xie et al. [140] reported alleviated stress levels especially under blue light. Similarly, chickens displayed lower fear responses (TI reaction) under UV light [135,136] or under green and blue light compared to red light [148] or white light [132]. Hence, light color may be an additional management tool to alleviate stress and fear responses in chickens, thereby improving immune functions.

### 5.3. Light Intensity

While there are several studies on the effect of light intensity on production parameters, relatively few studies have examined its effect on chickens’ immune system. Studying light intensities in broilers between 1 and 80 lux and under 10 lux or 25 lux in laying hens revealed lower H/L ratios at lower light intensities [132,149,150], with a positive linear relationship between the magnitude of light intensity and the H/L ratio [149]. Furthermore, a higher relative proportion of peripheral T lymphocytes [149] and higher specific serum antibody titers after IBD vaccination [151] was observed under lower light intensities (varying between 5 and 80 lux). Most other studies, however, did not find an influence of light intensity (5 lux–200 lux) on serum white blood cell counts [131] and peripheral lymphocyte proliferation after stimulation with PHA or LPS [152]. Neither was any impact on specific antibody response after KLH [152], or SRBC stimulation [153,154], after NDV vaccination [151] or in total serum IgY, IgM and IgA [126] recorded in boilers.

Similarly, no difference in TI reactions [153] and plasma CORT concentrations [154,155,156,157] could be found when broilers were housed under different light intensities. However, some reports found higher serum CORT concentrations in broilers kept under 30 lux compared to 10 lux [126] and higher serum CORT concentrations as well as higher TI duration in laying hens under 25 lux compared to 10 lux [132].

With these findings in mind, we can assume that low light intensities (above a certain threshold value) probably have no detrimental effects on the birds’ immune and stress system, although, so far, data are too scarce to draw general conclusions.

### 5.4. Effect of Light Regimes on the Immune System—Possible Mechanisms and Conclusions

In conclusion, light regimes modify the immune function in chickens and may be used as a tool to maintain or achieve appropriate immune competence. We must, however, keep in mind that only a few immune parameters were included in most reports, and that by far not all relevant parts of the immune system were systematically investigated. Moreover, the diurnal rhythm of immune cells was not considered. Conclusions on whether certain light conditions are beneficial must, therefore, be considered with caution at this point. Keeping this restriction in mind, current data nevertheless suggest that light regimes with longer phases of darkness (corresponding to natural conditions) exhibit more beneficial effects on the immune parameters investigated, whereas light intensity itself seems not to be a critical factor.

Which underlying mechanisms may be responsible for this effect? Melatonin sets the internal biological clock governing different diurnal and seasonal cycles or rhythms in various physiological systems in birds [158]. Melatonin production is stimulated during the scotophase and inhibited by light during the photophase [158]. Exogenous administration of melatonin was shown to increase lymphocyte proliferation [117,121,125,142,143,159] and leukocyte numbers [121,159] and to decrease production of pro-inflammatory cytokines in chickens [121,125]. Furthermore, melatonin addition to the feed decreased the H/L ratio and increased antibody titers after SRBC stimulation in heat-stressed broilers [122]. Thus, a relationship between melatonin and poultry immunity can be assumed [152] and seems to be an important link between light regimes and poultry health. Agapito et al. [160] reported that a peak level of melatonin is observed in chickens only after 4 h of darkness under a 12L:12D light program. Compared with LD conditions, SD conditions apparently increase serum or plasma melatonin levels in broilers [124,125,126,161], which could explain the beneficial effect on the immune system of chickens housed under SD conditions. Corresponding to the above-mentioned results, light intensity (10 vs. 30 lux) did not influence serum melatonin concentrations [126]. In contrast, green light was shown to promote melatonin secretion [124,142,162,163] by enhancing the expressions of positive clock genes and repressing the expressions of negative clock genes [162]. The pineal contains a special photosensitive pigment that is sensitive to short wavelengths [164,165]. This special feature (photopigment) may be one factor explaining the sensitivity of immune function to shorter wavelength in birds. However, short wavelengths must be presented at higher intensities in order to affect the hypothalamus, while long wavelengths directly penetrate the brain even at lower intensities and then reach the hypothalamus [166]. Whether melatonin acts directly on immune cells or rather represents a hormonal measurement of time altering other mediators of immune competence in chickens remains to be investigated.

Another possible mechanism by which light affects the immune system is via the action of stress hormones. In birds and mammals, melatonin administration is associated with reduced CORT secretion [121,125,167,168], the down-regulation of glucocorticoid receptors [169] and attenuated negative effects of glucocorticoids on the immune system [167,170,171]. Elevated stress hormone concentrations due to specific light regimes are, therefore, likely to impair immune functions. Future studies combining in-depth immunological and endocrine analyses should address this possible interaction.

As already mentioned, physical activity can positively affect the immune system [91]. It has been shown that the light program influences the level and diurnal pattern of the locomotor activity in chickens. Broilers reared under SD compared to LD conditions were shown to be more active over a 24 h cycle [128,172]. LD conditions reduce both quantity and quality of sleep by causing a lack of flock synchrony that increases interruption of sleep by other birds [172]. It was also shown that activity increases during photoperiods with higher light intensities [152].

Recently, it has also been demonstrated that the photoperiod affects cecal microbiota in chickens [98] and that gut microbiota influences behavior, physiology and immune system in chickens [173,174].

To conclude, light management could be an effective tool to modulate the immune response in chickens. However, the interplay of photoperiod, light color and light intensity is also important and must be further investigated.

## 6. Impact of Ammonia and Hydrogen Sulfide on the Immune System in Chickens

Air quality is an important factor influencing welfare in poultry, as harmful concentrations of gases like ammonia (NH_3_) and hydrogen sulfide (H_2_S) unavoidably develop in intensive production systems. Many studies show that NH_3_ and H_2_S adversely affect bird performance including growth rate, feed efficiency, carcass quality and susceptibility to diseases [175,176]. High concentrations of these gases also impair the nervous, respiratory and the cardiovascular system and affect animal behavior [175,176,177,178]. It is noteworthy that the avian respiratory system is unique among vertebrates, and that, consequently, research in mammals including humans cannot be directly applied to poultry [175]. NH_3_ emission is strongly affected by manure management, temperature or litter moisture, and therefore concentrations vary between housing systems [175]. As NH_3_ originates from the decomposition of nitrogen-containing manure, NH_3_ concentrations are generally higher in litter-based housing types [175]. The average NH_3_ concentration ranges from 3 to 12 parts per minute (ppm) in enriched cages and from 66 to 122 ppm in littered floor systems [179]. In European countries, the maximum tolerated NH_3_ concentration for all animals kept indoors is 20 ppm [180]. H_2_S likewise results from the degradation of liquid manure under anaerobic conditions. Concentrations in poultry production vary between 0 to 9 ppm in floor-based and 0 to 0.2 ppm in cage-systems [181].

The influence of NH_3_ or H_2_S on the immune system is summarized in Table 4 and Table 5. McFarlane and Curtis [182,183] showed in an early study that relative numbers of heterophils increased, while lymphocytes and basophils decreased in broilers exposed to 125 ppm NH_3_. Similarly, laying hens and broilers showed a higher H/L ratio under 30 ppm NH_3_ compared to fresh air [184]. Monocytes and eosinophils were not affected [183]. Wei et al. [185] found lower ConA- and LPS-induced peripheral lymphocyte proliferation when broilers were exposed to 70 mg/kg NH_3_ compared to 30 mg/kg, while Wang et al. [186] did not see an effect for lower NH_3_ concentrations (13, 26 or 52 ppm compared to fresh air). When compared to fresh air conditions, exposure to NH_3_ between 26 and 60 ppm decreased antibody titers after NDV vaccination [186] and total serum concentrations of IgY, IgM and IgA [184,186,187]. Furthermore IgA concentrations in duodenal mucosa decreased when exposed to 70 ppm compared to 30 ppm [188]. The effect of NH_3_ on antibody production was reported to be dependent on dose and duration of exposure. Decreased antibody concentrations after NDV vaccination were evident in broilers exposed to 52 ppm for 14 days and when exposed to 25 or 52 ppm for 21 days. No effects were reported after one week of NH_3_ exposure independent of NH_3_ concentration. Similarly, an effect of NH_3_ on total IgY, IgM and IgA could only be seen after an exposure to 52 ppm, but not to 13 or 26 ppm [186]. NH_3_ triggers inflammation in the trachea and spleen due to an increased mRNA expression of pro-inflammatory cytokines like IL-4 or IL-1β when broilers are exposed to 65 or 70 ppm NH_3_ [185,189]. Similarly, Zhou et al. [190] reported that lower NH_3_ concentrations of 15 to 35 ppm increase the levels of IL-1β, IL-6 and IL-10 in serum, trachea and ileum, resulting in an inflammatory response. The tracheal cytokines were positively correlated with ileal cytokine concentrations [190], suggesting a cross-talk between the respiratory and intestinal tract.

H_2_S also exhibits profound effects on the immune system. Similarly to NH_3_, 20 or 30 ppm H_2_S activated inflammatory responses due to an increase in pro-inflammatory cytokines like TNF-α, IFN-γ, IL-6, IL-8 and IL-17 and a decrease in anti-inflammatory cytokines like IL-2, IL-4 and IL-10 in blood, spleen, and the bursa of Fabricius [177,178,192]. Hu et al. [178] additionally reported higher mRNA expressions of IgY, IgM and IgA in the bursa of Fabricius.

Both gases, 60 ppm NH_3_ or 20 ppm H_2_S, decreased the relative weight of bursa, spleen and thymus [172,181] and resulted in nuclear debris in the bursa of Fabricius and thymus [178,191]. Only a few studies found no influence of NH_3_ on immune parameters like antibody concentrations and cytokine expression after exposing laying hens to 30 ppm NH_3_ compared to fresh air [184] or on lymphoid organ weights after exposing broilers to up to 52 ppm NH_3_ compared to fresh air [186] or 70 ppm compared to 30 ppm NH_3_ [185].

It is important to note that NH_3_, together with stressors like heat or coccidiosis, generally leads to additive effects. McFarlane et al. [183] found a linear effect on relative numbers of heterophils and lymphocytes when broilers were exposed to 125 ppm plus an additional stressor. Moreover, elevated concentrations of atmospheric NH_3_ over a one-month period are associated with a prolonged increase in serum CORT [193]. Furthermore, other components of the aerial environment such as temperature, humidity, dust and pathogens have negative consequences for the immune system in chickens and may interact with harmful gases [194,195].

Taken together, the data show that particularly high NH_3_ or H_2_S concentrations pose a threat to chickens’ health by dampening adaptive immune response and promoting inflammation.

### Effect of Ammonia and Hydrogen Sulfide on the Immune System—Possible Mechanisms and Conclusions

The toxicity of these harmful gases depends on exposure concentration and duration, genetic background and overall management [184,186]. The most profound effects of high concentrations of harmful gases can be seen in the respiratory system. High levels of NH_3_ (100 ppm) were demonstrated to cause changes in the tissue of the trachea, leading to a decrease in the effectiveness of the mechanical defense system and promoting the multiplication and manifestation of microbial pathogens [196]. Indeed, chickens exposed to NH_3_ exhibited an increased susceptibility to Newcastle disease virus [197] and *Escherichia coli* [196]. The inhalation of 0.4% (4000 ppm) H_2_S in 15 min led to death in chickens [198]. NH_3_ and H_2_S have detrimental effects on the respiratory system and may cause inflammation. Nevertheless, many earlier studies have exposed birds to extremely high concentrations of NH_3_ and H_2_S, far higher than are present in commercial poultry production.

## 7. Research Gaps and Recommendations

The housing environment has the potential to affect the immune system of chickens. Although the hitherto existing studies vary substantially in their design (with respect to standardized housing conditions, breeds or age groups, duration of experimental phase, differences in immune parameters tested), some trends can be deducted. In general, stressful conditions are usually associated with high circulating CORT concentrations and fearfulness and have a dampening effect on adaptive immune function. On the other hand, stressors may also stimulate innate immune functions. A main consequence of this shift is often a weakened immune response to antigenic challenges (e.g., vaccinations), and an increased inflammatory state. This condition, if persisting chronically, has detrimental effects for animal health and can be considered as a poor welfare indicator. Moreover, it should be kept in mind that environmental stressors or management factors are likely to interact in an additive manner in modulating the immune system. Consequently, housing environment and management should be based on the respective needs of the animals in order to keep the stress status low, maintain appropriate immune function, and to elicit a robust response to pathogens. This is a prerequisite for high productivity, health and welfare.

Based on the current state of the art, research gaps and recommendations for future research are evident. The immune system consists of many different components which are balanced and sometimes even counterregulated. Thus, future studies should mirror this complexity by analyzing a corresponding set of parameters for both innate and adaptive immunity. Obviously, a combination of different immune parameters is necessary for a reliable indication of immune status and strength of immune defense. Until recently, adequate methods assessing immune parameters in birds were rare due to birds’ hematological particularities and the lack of an immunological toolbox. However, in recent years, a wide array of modern immunological methods became available for the assessment of number and distribution of leukocytes as well as functional capacity of immune cell types on a single cell level. This makes it possible to cover innate and adaptive immunity, including their relevant cellular and humoral components. For sound conclusions with respect to immune functioning, the set of parameters should include (1) the phenotype and count of leukocytes and lymphocyte subsets assessed by flow cytometry. Altered numbers of lymphoid immune cells point to altered migration and homing patterns or even to apoptosis of cells, both factors impairing immune competence. Increased numbers of innate immune cells may indicate inflammatory responses. (2) These numeric measures should be complemented by functional tests including proliferation capacity of lymphocytes, phagocytic capability, and cytotoxicity of CTL and NK cells. (3) Determination of cytokines in circulation as well as after antigenic stimulation. The balance between pro-inflammatory and anti-inflammatory cytokines drives the type of immune response and under- or overproduction can lead to an insufficient or exaggerated immune response. (4) To test clinical relevance, these immunological measures should ideally be matched by tests analyzing antibody response to vaccines and to novel antigens such as KLH or by challenge studies with disease models. Moreover, as most immune responses associated with vaccination are also controlled by TH cells and CTL, the memory function of T lymphocytes due to priming could be tested by measuring their specific responsiveness during vaccination or novel antigens through booster vaccination. These latter studies are useful because they assess the efficiency of coordinated and integrated immune processes to a relevant challenge.

Another set of knowledge gaps exists in the understanding of basic underlying physiological mechanisms in birds. This would help to understand how housing conditions and management factors, alone and in combination, affect the bidirectional relation of the immune and neuroendocrine system. Better understanding would allow us to shape a housing environment according to the needs of the animals and targeted interventions (e.g., nutritional manipulation) to avoid allostatic overload leading to stress, modified immune response and potentially enhanced disease susceptibility.

The same also applies to effects of early life conditioning on immune competence in chickens. The early life period is the period when the animal is most sensitive to environmental conditions, due to high plasticity in the developing brain [199]. Glucocorticoid exposure to the developing brain induces alterations in gene expression, and causes a hyperresponsive HPA axis and increased anxiety behavior. Moreover, the immune function of the late embryonic and neonatal chickens is also not yet entirely developed and undergoes age-dependent variations in respect of structure and occurrence of lymphoid organs as well as immune cell distribution [200,201,202,203]. It is well known that early life experiences have long-term effects on physiology and behavior later in life in poultry [204,205]. Thus, housing environment during the early life stages could modify chickens’ immune system for better or worse in later life stages, which is especially interesting for laying hens and hence requires further research. The age at the time of stressor exposure, as well as duration and type of the stressor, plays a decisive role in how the physiological and behavioral response is affected and appears important for the long-term consequences.

In addition to age, the genetic background may have a profound impact on how chickens deal with their housing environment and how their immune system reacts. Some strains of chicken are generally considered to be more fearful than others and show a higher stress response to environmental stimuli [62]. Layers and broilers were also shown to differ regarding the distribution of immune cells and strength of cellular and humoral immune response [206,207,208]. Thus, selective breeding presents an interesting, complementary possibility to increase vaccine efficacy and disease resistance by using heritable traits like the concentration of natural antibodies [209]. Moreover, there is an impact of the genetic background on gut microbiota composition [210,211] that is clearly associated with host stress response and a mediator of host health. Even though the gut microbiota of chickens has received much attention in recent years and was demonstrated to be influenced by housing environment [98,210,212], there is still a lack of information on how the microbiota interacts with the host immune system. Nevertheless, the high plasticity of the avian microbiome offers a good basis to intentionally manipulate the microbiota by nutrition or housing condition to improve intestinal barrier function and host immunity [213].

So far, drawing general conclusions for practical application is often hampered by a lack of standardized measures across studies, the use of different breeds and age groups, or variation in the severity and duration of the stressor. Obviously, there is also urgent need for the use of a standardized set of immunological parameters as suggested above. This would allow for a better comparability of immunological results across studies, and would be most useful for the application of results to commercial poultry production. Nevertheless, some obvious trends and recommendations can already be deducted from current research, as some housing conditions appear clearly less favorable for immune competence in poultry. However, again, more in-depth immunological analysis under standardized conditions is required to confirm and extend current knowledge.

In summary, the understanding of immunosuppressive risk factors is essential for successful poultry management aiming to optimize health, welfare and efficiency. Chronic stressful conditions alter biological functions, disrupt homeostasis and, therefore, reduce the immune response to vaccinations or pathogens, thus increasing disease susceptibility during poultry production. Including the immune system in the research on the impact of housing environment on chickens is a prerequisite for sustainable poultry production optimized on an economic, social and environmental level.

## Figures and Tables

**Table 1:** Alterations of immune parameters of chickens housed in conventional cages (CC) compared to enriched cages (EC).

Immune Parameter	Sample	CC vs. EC	Reference
Total leukocytes	Blood	↔	[70]
% of total leukocytes			
Heterophils (H)	Blood	↑	[70,71,72]
		↔	[73,74]
Total lymphocytes (L)	Blood	↓	[70,71,72]
		↑	[74]
		↔	[73]
T helper cells	Blood	↔	[70,71]
Cytotoxic T cells	Blood	↔	[70,71]
Monocytes	Blood	↑	[72]
		↔	[70,73,74]
Basophils	Blood	↔	[70,72,73,74]
Eosinophils	Blood	↑	[72]
		↔	[70,73,74]
H/L ratio	Blood	↑	[71,72,73,75]
		↔	[70]
Functionality of monocytes			
Chemotaxis	Blood	↔	[75]
Phagocytosis	Blood	↔	[75]
Functionality of heterophils			
Chemotaxis	Blood	↔	[70,71]
Phagocytosis	Blood	↔	[70,71]
Oxidative burst	Blood	↔	[70,71]
Antibody concentration			
IgY	Blood	↔	[76]
Newcastle disease virus	Blood	↔	[72,74,75]
Infectious bronchitis virus	Blood	↔	[72]
Sheep red blood cells	Blood	↓	[70,71]
Relative organ weight			
Bursa of Fabricius		↔	[70,71]
Thymus		↔	[70,71]
Spleen		↔	[70,71]

↔ = no difference, ↑ = increased/higher, and ↓ = decreased/lower; Ig = immunoglobulin.

**Table 2:** Alterations of immune parameters of chickens provided with constant light under long-day conditions (LD-CL) compared to short-day conditions (SD-CL).

Immune Parameter	Sample	LD-CL vs. SD-CL	Reference
Total leukocytes	Blood	↑	[111]
% of total leukocytes			
Heterophils (H)	Blood	↑	[112,113]
		↔	[115,116]
Total lymphocytes (L)	Blood	↓	[112,113]
		↔	[115,116]
Monocytes	Blood	↓	[112]
Basophils	Blood	↓	[112]
Eosinophils	Blood	↓	[112]
T lymphocytes	Spleen	↓	[117]
T helper cells	Spleen	↓	[117]
Cytotoxic T cells	Spleen	↓	[117]
B lymphocytes	Spleen	↔	[117]
H/L ratio	Blood	↑	[112,113,114]
		↓	[111]
		↔	[115,116]
Functionality of lymphocytes			
Proliferation to pokeweed mitogen	Blood	↔	[111,117]
	Spleen	↓	[117]
Proliferation to concanavalin A	Blood, spleen	↔	[111,117]
Cell-mediated immunity			
Delayed-type hypersensitivity to phytohemagglutinin	Blood	↓	[118]
Delayed-type hypersensitivity to concanavalin A	Blood	↓	[118]
Antibody concentration			
Newcastle disease virus	Blood	↔	[114]
Sheep red blood cells	Blood	↓	[118]
		↔	[103,111,114,119]
Relative organ weight			
Bursa of Fabricius		↔	[114,120]
Spleen		↔	[114]

↔ = no difference, ↑ = increased/higher, and ↓ = decreased/lower.

**Table 3:** Alterations of immune parameters in chickens housed under long-day conditions with constant lighting (LD-CL) compared to short-day conditions with intermittent lighting (SD-IML).

Immune Parameter	Sample	LD-CL vs. SD-IML	Reference
Total leukocytes	Blood	↓	[111,121]
% of total leukocytes			
Heterophils (H)	Blood	↑	[112]
Total lymphocytes (L)	Blood	↓	[112]
Monocytes	Blood	↓	[112]
Eosinophils	Blood	↔	[112]
Basophils	Blood	↔	[112]
T lymphocytes	Blood, spleen	↓	[117,127]
T helper cells	Spleen	↓	[117]
Cytotoxic T cells	Spleen	↓	[117]
B lymphocytes	Spleen	↔	[117]
H/L ratio	Blood	↑	[112]
		↔	[111,122,123]
Functionality of lymphocytes			
Proliferation to pokeweed mitogen	Blood, spleen	↓	[111,117]
Proliferation to concanavalin A	Blood, spleen	↓	[111,117,125]
Proliferation to pokeweed mitogen	Blood	↔	[117]
Proliferation to concanavalin A	Blood	↔	[117,121]
Functionality of monocytes			
Phagocytosis	Blood	↓	[124]
Functionality of basophils			
Delayed-type hypersensitivity to phytohemagglutinin	Blood	↔	[125]
Antibody concentration			
IgM	Blood	↓	[126]
IgY	Blood	↔	[126]
		↓	[127]
IgA	Blood	↔	[126]
Newcastle disease virus	Blood	↓	[123]
Sheep red blood cells	Blood	↔	[103,122]
		↓	[111]
Cytokine concentration			
IL-6	Blood	↔	[121,125]
Relative organ weight			
Spleen		↔	[124,127]
Bursa of Fabricius		↓	[124]
		↔	[123,127]
Thymus		↓	[124]
		↔	[123,127]

↔ = no difference, ↑ = increased/higher, and ↓ = decreased/lower; Ig = immunoglobulin; IL = interleukin.

**Table 4:** Alterations of immune parameters of chickens exposed to ammonia (NH_3_) (treatment) compared to control groups.

Parameter	Sample	Treatment vs. Control	Reference
% of total leukocytes			
Heterophils (H)	Blood	↑	[183]
Total lymphocytes (L)	Blood	↓	[183]
Monocytes	Blood	↔	[183]
Basophils	Blood	↓	[183]
Eosinophils	Blood	↔	[183]
H/L ratio	Blood	↑	[182,183,184]
Functionality of lymphocytes			
Proliferation to concanavalin A	Blood	↓	[185]
		↔	[186]
Proliferation to lipopolysaccharide	Blood	↓	[185]
		↔	[186]
Antibody concentration			
IgM	Blood	↓	[184,186,187]
IgY	Blood	↓	[186,187]
	Blood	↔	[184]
IgA	Blood, duodenum	↓	[186,187,188]
	Blood	↔	[184]
Newcastle disease virus	Blood	↓	[186]
Cytokine concentration			
IL-1β	Spleen, trachea	↑	[185,189,190]
	Blood, spleen	↔	[184]
IL-2	Trachea	↑	
IL-4	Spleen, trachea	↑	[185,189]
Il-6	Spleen	↔	[184,190]
	Trachea	↑	[189]
IL-10	Trachea	↑	[189,190]
IL-17	Trachea	↑	[189]
IFN-γ	Blood	↔	[184]
	Trachea	↓	[189]
TNF-α	Trachea	↑	[189]
	Blood, spleen	↔	[184]
Relative organ weight			
Spleen		↓	[187]
		↔	[185,186]
Thymus		↓	[187,191]
		↔	[185,186]
Bursa		↓	[187,191]
		↔	[185,186]

↔ = no difference, ↑ = increased/higher, and ↓ = decreased/lower; Ig = immunoglobulin; IL = interleukin; IFN = interferon; TNF = tumor necrosis factor.

**Table 5:** Alterations of immune parameters of chickens exposed to hydrogen sulfide (H_2_S) (treatment) compared to control groups.

Parameter	Sample	Treatment vs. Control	Reference
Antibody concentration			
IgM	Bursa of Fabricius	↓	[178]
IgY	Bursa of Fabricius	↓	[178]
IgA	Bursa of Fabricius	↓	[178]
Newcastle disease virus	Blood	↓	[178]
Cytokine concentration			
IL-1β	Blood, bursa of Fabricius, spleen	↑	[177,178,192]
IL-2	Blood	↓	[177]
IL-4	Blood, bursa of Fabricius	↓	[177,178]
Il-6	Blood, bursa of Fabricius	↑	[177,178]
Il-8	Blood	↑	[177]
IL-10	Blood, bursa of Fabricius	↓	[177,178]
IL-12	Blood, bursa of Fabricius	↑	[177,178]
IFN-γ	Bursa of Fabricius	↑	[178]
	Blood	↓	[177]
TNF-α	Blood, bursa of Fabricius, spleen	↑	[177,178,192]
Relative organ weight			
Bursa of Fabricius		↔	[178]

↔ = no difference, ↑ = increased/higher, and ↓ = decreased/lower; Ig = immunoglobulin; IL = interleukin; IFN = interferon; TNF = tumor necrosis factor.
